# Convergent ablation for persistent atrial fibrillation: outcomes from a single-centre real-world experience

**DOI:** 10.1093/ejcts/ezac515

**Published:** 2022-11-08

**Authors:** Nilanka N Mannakkara, Bradley Porter, Nicholas Child, Baldeep S Sidhu, Vishal S Mehta, Mark K Elliott, Justin Gould, Shahada Ahmed, Reza Razavi, Christopher A Rinaldi, Christopher Blauth, Jaswinder S Gill

**Affiliations:** Department of Cardiovascular Services, Guy’s and St. Thomas’ Hospital, London, UK; School of Biomedical Engineering and Imaging Sciences, King’s College London, London, UK; School of Biomedical Engineering and Imaging Sciences, King’s College London, London, UK; School of Biomedical Engineering and Imaging Sciences, King’s College London, London, UK; School of Biomedical Engineering and Imaging Sciences, King’s College London, London, UK; Department of Cardiovascular Services, Guy’s and St. Thomas’ Hospital, London, UK; School of Biomedical Engineering and Imaging Sciences, King’s College London, London, UK; Department of Cardiovascular Services, Guy’s and St. Thomas’ Hospital, London, UK; School of Biomedical Engineering and Imaging Sciences, King’s College London, London, UK; Department of Cardiovascular Services, Guy’s and St. Thomas’ Hospital, London, UK; School of Biomedical Engineering and Imaging Sciences, King’s College London, London, UK; Department of Cardiovascular Services, Guy’s and St. Thomas’ Hospital, London, UK; School of Biomedical Engineering and Imaging Sciences, King’s College London, London, UK; Department of Cardiovascular Services, Guy’s and St. Thomas’ Hospital, London, UK; School of Biomedical Engineering and Imaging Sciences, King’s College London, London, UK; Department of Cardiovascular Services, Guy’s and St. Thomas’ Hospital, London, UK; Department of Cardiovascular Services, Guy’s and St. Thomas’ Hospital, London, UK; School of Biomedical Engineering and Imaging Sciences, King’s College London, London, UK

**Keywords:** Atrial fibrillation, Catheter ablation, Hybrid ablation, Persistent atrial fibrillation, Arrhythmias

## Abstract

**OBJECTIVES:**

Atrial fibrillation (AF) is common and can cause significant morbidity and detriment to quality of life. Success rates for conventional catheter ablation are suboptimal in persistent AF (PsAF), especially when longstanding. Convergent hybrid ablation combines endoscopic surgical epicardial and endocardial catheter ablation. It offers promise in treating PsAF. We aimed to evaluate outcomes at our centre following convergent ablation.

**METHODS:**

We conducted an observational study of patients undergoing ablation from 2012 to 2019 at a London cardiac centre. Sixty-seven patients underwent convergent ablation entailing epicardial ablation, mostly via sub-xiphoid access, followed by endocardial left atrial catheter ablation. Baseline and follow-up data were obtained retrospectively from clinical records. Primary outcome was freedom from AF on/off anti-arrhythmic drugs after 12-month follow-up. Secondary outcomes included freedom from AF over the entire follow-up, freedom from anti-arrhythmic drugs, freedom from atrial arrhythmias, symptom status, repeat ablation and complications.

**RESULTS:**

At baseline, 80.6% had PsAF >1 year (80.6%), 49.3% had body mass index >30 kg/m^2^ at baseline and 19.4% had left ventricular ejection fraction of 40% or less. The median follow-up was 2.3 (1.4–3.7) years. Freedom from AF recurrence was 81.3% at 1 year and 61.5% over overall follow-up. Eleven patients (16.4%) required redo AF ablation. Prolonged AF duration was associated with increased recurrence at 12 months and duration >5 years with a shorter time to recurrence on Kaplan–Meier analysis, but this and other factors did not significantly impact the AF recurrence during the overall follow-up period.

**CONCLUSIONS:**

Convergent ablation had good 1-year and overall success rates for treating PsAF. Our results in a diverse, real-world population support the potential of convergent ablation in patients with challenging to treat PsAF.

**Figure:**
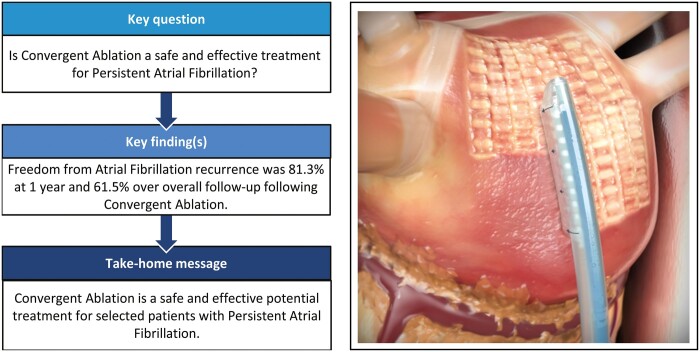

## INTRODUCTION

Atrial fibrillation (AF) is the commonest arrhythmia encountered in clinical practice, affecting 33.5 million people globally, with an increasing worldwide disease burden [1–3]. AF causes significant morbidity, healthcare and economic burden and is associated with increased risk of stroke, heart failure, cognitive decline, hospitalization and death [2].

Pulmonary vein isolation (PVI) through catheter ablation is effective for treating paroxysmal AF. In contrast, persistent AF (PsAF) ablation success rates are suboptimal, especially when longstanding. There is no widespread consensus on the optimal ablation strategy and outcomes vary greatly in the literature. Some reported success rates are as low as 20% from a single procedure [4]. A treatment gap exists; PsAF patients comprise a significant proportion of the AF population and can experience debilitating symptoms and impaired QOL.

PsAF may be sustained by numerous extrapulmonary vein drivers and substrates involving the posterior wall, including focal ectopic triggers, rotors and complex fractionated atrial electrograms. Creating durable, transmural endocardial posterior wall lesions to augment PVI by catheter ablation is challenging, even with multiple procedures. Several studies have failed to conclusively show an incremental benefit of adding such additional lesions to PVI [5–7].

Convergent ablation is a hybrid method that combines minimally invasive endoscopic surgical epicardial ablation with endocardial catheter ablation in 2 stages [8]. It reduces the risks of oesophageal and phrenic nerve injury and allows direct left atrium (LA) visualization whilst being less invasive than full surgical ablation. Epicardial ablation can be performed via a small incision and without the need for sternotomy or cardiopulmonary bypass. Epicardial fat and ganglionic plexi, which may also help to sustain PsAF, can also be targeted.

Initial analyses of the convergent procedure and a recent randomized controlled trial have shown promising results on its effectiveness in treating PsAF [9–12]. Our centre was the first in the UK to perform the convergent procedure. We investigated our cohort of patients with the aim of evaluating procedure outcomes and further understanding which patients may be best served by this approach.

## METHODS

### Study design

All patients who underwent a two-stage convergent ablation procedure for PsAF between 2012 and 2019 at a single UK tertiary centre (St. Thomas’ Hospital, London) were included in our analysis. The study received institutional approval.

Patients with PsAF with indications for convergent ablation were referred by their electrophysiologist. Generally, this was defined as patients with an indication for AF ablation but considered to have poor likelihood of success from conventional catheter ablation. Our centre does not run a routine surgical AF ablation program (e.g. Cox-maze procedure), though this can be performed in selected cases or when undergoing concomitant cardiac surgery. Patients were required to have either trialled or be unsuitable for a class I/III anti-arrhythmic medication. Those with contraindications to anticoagulation or prohibitive surgical risk were excluded and not considered for convergent ablation. Outcome and follow-up data were obtained retrospectively from the notes and clinical database.

### Convergent ablation

**Figure 1:**
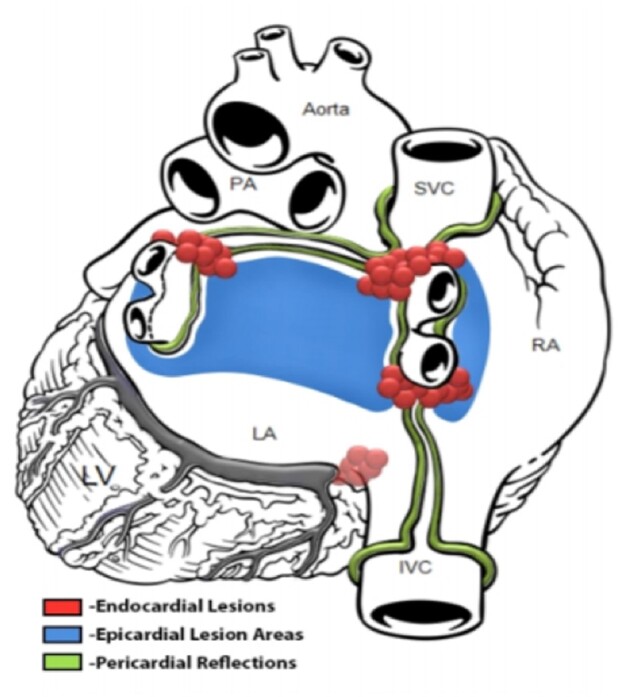
Example of a combined lesion set from both stages of the convergent ablation procedure.

All patients underwent a two-stage convergent procedure. Epicardial ablation (first stage) was performed by a cardiac surgeon under general anaesthetic with standard ventilation. Surgical convergent ablation technique has previously been described in the literature [8]. The posterior pericardial space was accessed via a small transdiaphragmatic incision or sub-xiphoid access. A cannula was inserted into the oblique sinus allowing direct visualization of the posterior LA using an endoscope. A saline-irrigated EPIsense Visitrax radiofrequency ablation catheter (Atricure, USA) was inserted alongside and used to deliver sequential and continuous parallel vertical connecting ablation lesions to the posterior left atrial wall with the aim of ablating the entire posterior surface (see Fig. 1 for example of lesion sets). Partial PVI was also performed. Lesion completeness was assessed by direct visualization to assess interconnectedness and using sensing electrodes to assess impedance. Once ablation was complete, a pericardial drain was usually inserted for 24 h post-procedure.

Following this, endocardial catheter ablation (second stage) was performed under general anaesthesia at one of 2 intervals: (i) same sitting—immediately after the first stage or (ii) as a staged procedure—after ∼6–12 weeks. The LA was accessed using a standard approach via femoral venous access and transseptal puncture. Electroanatomical voltage mapping was performed using CARTO3 (Biosense Webster, USA) or Ensite NavX (Abbott, USA) mapping system. PVI was completed, with pulmonary vein silencing confirmed by voltage mapping. Further ablation lesion sets [such as further posterior wall ablation or cavotricuspid isthmus (CTI) line] were performed at the discretion of the operator and according to the completeness of posterior wall isolation observed on endocardial voltage mapping. Electrical cardioversion was performed if patients were not in sinus rhythm at the end of the procedure.

### Follow-up

Patients were reviewed in the electrophysiology clinic ∼8–12 weeks post-discharge. Rhythm status at the first appointment was assessed by ambulatory electrocardiogram (ECG) monitor (1-day ambulatory ECG monitor or more) (in the majority of patients) or 12-lead ECG. Continuous rhythm monitoring at subsequent follow-up was performed according to symptoms and physician discretion. If patients experienced significant symptoms but had a normal resting ECG at the time of follow-up, further continuous rhythm monitoring was sought. Recurrence of AF or atrial arrhythmia (AA) was defined as documented arrhythmia on ECG or continuous rhythm monitor, lasting at least 30 s. A 3-month blanking period post-catheter ablation was used.

The primary outcome was freedom from AF on/off anti-arrhythmic drugs (AADs) at 1-year follow-up. Secondary outcomes were overall freedom from AF (defined as over the overall course of follow-up) and at 1-year off AADs, freedom from AA, requirement for repeat ablation and 1-year symptom status [as assessed by modified EHRA (mEHRA) scale].

### Statistical analysis

Normally distributed variables are presented as mean [standard deviation (SD)]. Non-normally distributed or skewed variables are presented as median with interquartile range. Kaplan–Meier analysis was used for time-to-event analyses, with log-rank test for the comparison of groups. Cox regression analysis, specifying a 95% confidence interval, was used to assess for predictors of outcome. Following the univariable analysis, all variables were entered into a multivariable cox regression model, and non-significant variables were sequentially eliminated using a backward elimination approach (with exit criteria of *P* = 0.15) to select variables for use in the final model. A *P*-value of <0.05 was considered statistically significant for all tests. Statistical analyses were performed using SPSS version 28 (IBM Corporation, Switzerland).

## RESULTS

Sixty-seven patients underwent a two-stage convergent ablation and were included in the analysis. Five other patients underwent the first stage only and so were excluded from further analysis. The reasons for not completing the second stage included: loss to follow-up (*n* = 1), diagnosis of malignancy causing deferral/cancellation (*n* = 2) and death (*n* = 2, deemed unrelated to procedure). The median follow-up was 2.3 (1.4–3.7 years). The mean follow-up was 2.8 years.

Baseline characteristics are seen in Table 1. The mean age was 61.7 (SD: 11.3) years and the majority were male (88.1%). The indication for ablation in all patients was PsAF. The mean AF duration was 3.2 (SD: 2.7) years, 80.6% had an AF duration >1 year and 14.9% had AF for longer than 5 years. 38.8% had undergone previous AF ablation. Of those patients, the average number of previous AF ablation procedures was 2.1 (SD: 1.4).

**Table 1:** Baseline characteristics and medications

Characteristic	
Age (years)	61.7 (SD: 11.3)
Male	59 (88.1%)
Previous failed AF ablation	26 (38.8%)
AF duration (years)	3.2 (SD: 2.7)
AF duration >1 year	54 (80.6%)
LA diameter	45.6 mm (SD: 8.0)
LA diameter >50 mm	20 (29.9%)
Left ventricular ejection fraction	49.1% (SD: 9.4)
Left ventricular ejection fraction ≤40%	13 (19.4%)
Body mass index	32.1 kg/m^2^ (SD: 5.8)
Body mass index >30 kg/m^2^	33 (49.3%)
Hypertension	26 (38.8%)
Diabetes	12 (17.9%)
Obstructive sleep apnea	7 (11.9%)
Dilated cardiomyopathy	7 (11.9%)
Previous history of alcohol excess	4 (6.0%)
Medications at baseline	
Beta-blocker	66 (98.5%)
Class I AAD	7 (10.4%)
Class III AAD	25 (37.3%)

Baseline characteristics and medications [values are expressed as mean (SD), or *n* (%)].

AAD: anti-arrhythmic drug; AF: atrial fibrillation; LA: left atrium; SD: standard deviation.

**Figure 2:**
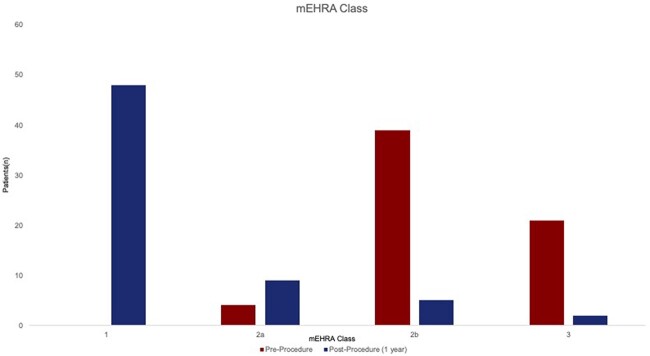
Modified EHRA classification pre- versus post-procedure—proportion of patients in each modified EHRA class before procedure and at 1 year post-procedure follow-up.

The mean left ventricular ejection fraction (LVEF) was 49.1%. 19.4% patients had LVEF ≤40%. The mean LA diameter was 45.6 (SD: 8.0) mm, with 29.9% of patients having LA diameter >50 mm. 49.3% had body mass index >30 kg/m^2^. The majority of patients reported symptoms consistent with mEHRA class IIb (moderate symptoms troubling patient)/III (severe symptoms affecting daily activity) (Fig. 2).

### Procedure details

One patient underwent conversion to median sternotomy during the epicardial procedure due to multiple pericardial adhesions limiting access for complete epicardial ablation. 34.3% underwent the two-stage procedure in a single sitting; 65.7% had a staged approach. Catheter ablation lesion sets performed are seen in Tables 2 and 3. PVI was performed in all but 2 patients, who already had silent pulmonary veins. None of the patients with PsAF duration <1 year received additional ablation lesions above PVI and CTI lines.

**Table 2:** Ablation strategy

Ablation lesion	Patients
Pulmonary vein isolation, *n* (%)	63 (96.9) (1 cryoablation; 62 RFA)
Cavotricuspid isthmus ablation, *n* (%)	14 (21.5)
Complex fractionated atrial electrogram ablation, *n* (%)	3 (4.6)
Left atrial roof line, *n* (%)	9 (13.8)
Mitral line, *n* (%)	7 (10.8)
Posterior box, *n* (%)	4 (6.2)
Other LA lines/lesions, *n* (%)	13 (20.0)

Lesion sets are performed during catheter ablation (lesion set data are unavailable for 2 patients).

LA: left atrium; RFA: radiofrequency ablation.

**Table 3:** Ablation strategy according to persistent atrial fibrillation duration less or more than 1 year

Ablation lesion	PsAF duration <1 year	PsAF duration >1 year
	(*n* = 10)	(*n* = 55)
Pulmonary vein isolation, *n* (%)	10 (100.0)	53 (96.4) (1 cryoablation; 52 RFA)
Cavotricuspid isthmus ablation, *n* (%)	2 (20.0)	12 (21.8)
Complex fractionated atrial electrogram ablation, *n* (%)	0 (0.0)	3 (5.5)
Left atrial roof line, *n* (%)	0 (0.0)	9 (16.4)
Mitral line, *n* (%)	0 (0.0)	7 (12.7)
Posterior box, *n* (%)	0 (0.0)	4 (7.2)
Other LA lines/lesions, *n* (%)	0 (0.0)	13 (23.6)

Lesion sets are performed during catheter ablation (lesion set data are unavailable for 2 patients).

LA: left atrium; PsAF: persistent atrial fibrillation; RFA: radiofrequency ablation.

### Outcomes

**Figure 3:**
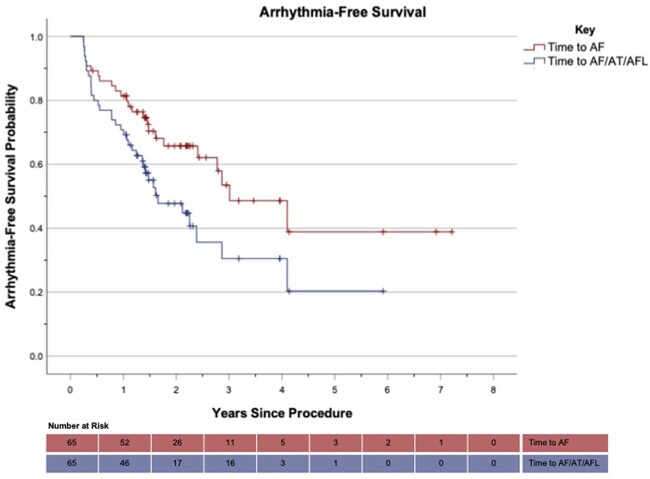
Arrhythmia-free survival in patients undergoing convergent procedure (time-to-recurrence Kaplan–Meier analysis) (‘+’ denotes censored event).

Fifteen (22.4%) patients required direct current cardioversion (DCCV) during the blanking period. Two patients were not evaluable post-blanking period (death of unknown cause at 3 months, *n* = 1; loss to follow-up, *n* = 1). 81.3% had freedom from AF recurrence on/off AADs and 68.8% had freedom from AF recurrence and were off AADs (excluding 1 patient who had been lost to follow-up without experiencing AF recurrence post-blanking period but before 1 year) (see Tables 4 and 5). One-year freedom from any AA was 69.2%. Overall freedom from AF recurrence was 61.5% over the entire follow-up period. Time-to-recurrence analysis for AF/AA is seen in Fig. 3. The majority of patients (75.0%) were classified as mEHRA class 1 (asymptomatic) at 1 year post-ablation (Fig. 2).

**Table 4:** Outcomes following convergent ablation

Outcome	
Freedom from AF recurrence on/off AADs at 1 year, % (*n*)	81.3 (52)
Freedom from AF recurrence and off AADs at 1 year, % (*n*)	68.8 (44)
Freedom from AF/AT/AFL at 1 year, % (*n*)	69.2 (45)
mEHRA class 1 (asymptomatic) at 1 year, % (*n*)	75.0 (48)
Overall freedom from AF recurrence, % (*n*)	61.5 (40)
Overall freedom from AF/AT/AFL, % (*n*)	44.6 (29)
Incidence of AT/AFL post-blanking period, % (*n*)	30.8 (20)
DCCV required in blanking period, % (*n*)	22.4 (15)
DCCV required post-blanking period, % (*n*)	30.8 (20)
Repeat AF ablation, % (*n*)	16.9 (11)
Repeat ablation for AT/AFL only, % (*n*)	12.3 (8)

AADs: anti-arrhythmic drugs; AF: atrial fibrillation; AFL: atrial flutter; AT: atrial tachycardia; DCCV: direct current cardioversion; mEHRA: modified EHRA.

**Table 5:** Arrhythmia following convergent ablation in subgroups

Outcome	PsAF duration >1 year	LA diameter >50 mm	LVEF ≤40%	BMI >30 kg/m^2^	Previous AF ablation
	(*n* = 52)	(*n* = 19)	(*n* = 12)	(*n* = 33)	(*n* = 26)
AF recurrence at 1 year, % (*n*)	17.3 (9)	26.3 (5)	16.7 (2)	18.8 (6)	15.4 (4)
Overall AF recurrence, % (*n*)	38.5 (20)	63.2 (12)	50.0 (6)	27.3 (9)	46.2 (12)
Overall occurrence of AF/AT/AFL, % (*n*)	51.9 (27)	73.7 (14)	50.0 (6)	45.5 (15)	61.5 (16)

AF: atrial fibrillation; AFL: atrial flutter; AT: atrial tachycardia; BMI: body mass index; LA: left atrium; LVEF: left ventricular ejection fraction; PsAF: persistent atrial fibrillation.

A total of 69.2% of patients underwent a form of continuous ECG monitoring at follow-up (at least 1-day Holter ECG monitor and including 2 who had pacemakers pre-procedure). In these patients, AF recurrence at 1 year was 13.3%, overall AF recurrence was 37.8% and overall AA recurrence was 48.9%. In those who did not undergo continuous monitoring (31.8%), AF recurrence at 1 year was 31.5%, overall AF recurrence over follow-up was 40.0% and overall AA recurrence was 65.0%. Of those who did not undergo continuous monitoring, some already had a 12-lead ECG confirming recurrence and did not undergo further continuous ECG monitoring.

Nineteen patients required repeat ablation. Of these, 8 patients had ablation for atrial tachycardia (AT)/atrial flutter (AFL) alone and 11 patients required repeat AF ablation. Twenty patients required DCCV post-blanking period. Four patients underwent subsequent AV node ablation with pacemaker implantation due to the recurrence of AF, of whom 2 had undergone unsuccessful repeat AF ablation.

Twelve patients with AFL/tachycardia proceeded to repeat ablation, 4 combined with AF ablation and 8 had ablation for AFL/AT only. The following was found: 3 patients had right-sided CTI-dependent flutter, 8 patients had a LA tachycardia (peri-mitral flutter/tachycardia: 4, adjacent to a pulmonary vein: 2, posterior wall: 1, unspecified: 1) and in 1 patient, no tachycardia was inducible during the study.

### Predictors of recurrence

Prolonged baseline PsAF duration was associated with AF recurrence at 12 months [hazard ratio (HR) 1.19, 95% confidence interval (CI) = 1.01–1.40, *P* = 0.04]. Other factors did not show association with 12-month AF recurrence (Table 6). However, PsAF duration was not significantly associated with overall AF recurrence over the course of follow-up (HR 1.09, 95% CI 0.97–1.23, *P* = 0.14). No individual characteristics were significantly predictive of overall AF recurrence on univariable analysis or multivariable analysis, though LA diameter trended towards significance (HR 1.05, 95% CI 1.00–1.11, *P* = 0.05).

**Table 6:** Predictors of arrhythmia recurrence

Feature	*P*-Value	Hazard ratio	95% confidence interval
12-Month AF recurrence			
Univariable analysis			
Age (years)	0.21	1.04	0.98–1.09
Gender (male)	0.60	0.66	0.15–3.02
Previous ablation	0.55	0.70	0.21–2.31
PsAF duration (years)	0.04^*^	1.19	1.01–1.40
LA diameter (mm)	0.29	1.04	0.97–1.12
EF (%)	0.61	1.02	0.95–1.10
BMI (kg/m^2^)	0.65	0.97	0.87–1.09
Diabetes	0.54	1.51	0.41–5.58
Hypertension	0.88	1.09	0.35–3.45
Two-stage procedure	0.42	1.72	0.47–6.34
Overall AF recurrence			
Univariable analysis			
Age (years)	0.08	1.03	1.00–1.07
Gender (male)	0.81	0.87	0.29–2.59
Previous ablation	0.70	1.17	0.53–2.57
PsAF duration (years)	0.14	1.09	0.97–1.23
LA diameter (mm)	0.05	1.06	1.00–1.11
EF (%)	0.36	0.98	0.94–1.02
BMI (kg/m^2^)	0.28	0.96	0.88–1.04
Diabetes	0.98	0.99	0.37–2.65
Hypertension	0.79	1.11	0.51–2.46
Two-stage procedure	0.46	1.40	0.58–3.35
Multivariable analysis			
LA diameter	0.05	1.05	1.00–1.11
Age (years)	0.09	1.03	0.99–1.07
Overall AA recurrence			
Univariable analysis			
Age (years)	0.34	1.01	0.99–1.04
Gender (male)	0.84	1.11	0.41–2.98
Previous ablation	0.34	1.38	0.71–2.67
PsAF duration (years)	0.18	1.08	0.96–1.22
LA diameter (mm)	0.14	1.03	0.99–1.08
EF (%)	0.81	1.00	0.96–1.03
BMI (kg/m^2^)	0.33	0.97	0.91–1.03
Diabetes	0.60	0.79	0.33–1.90
Hypertension	0.15	0.60	0.30–1.20
Two-stage procedure	0.07	2.00	0.94–4.27
Multivariable analysis			
Previous ablation	0.08	1.85	0.93–3.66
LA diameter (mm)	0.08	1.04	1.00–1.09
Hypertension	0.16	0.60	0.30–1.23
Two-stage procedure	0.04^*^	2.24	1.05–4.80

N.B. for assessment of 12-month AF recurrence, only PsAF duration met criteria for inclusion in the multivariable model and therefore further multivariable analysis was not performed for this outcome.

AA: atrial arrhythmia; AF: atrial fibrillation; BMI: body mass index; EF: ejection fraction; LA: left atrium; PsAF: persistent atrial fibrillation.

* denotes *p*-value < 0.05.

**Figure 4:**
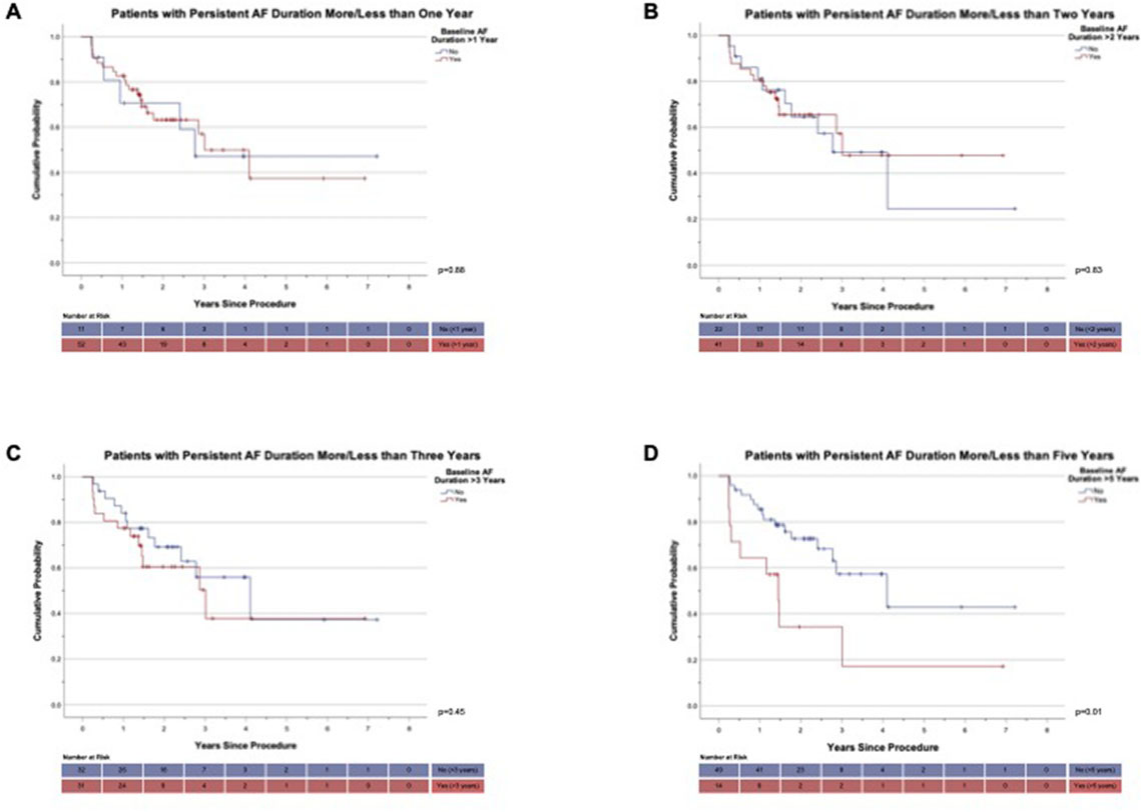
Atrial fibrillation-free survival probability according to persistent atrial fibrillation duration [time to recurrence when patients categorized based on baseline persistent atrial fibrillation duration greater or less than 1 year (**A**), 2 years (**B**), 3 years (**C**) or 5 years (**D**)] (‘+’ denotes censored event).

Those who had a baseline AF duration of >5 years had a significantly shorter time to AF recurrence (*P* = 0.01) on Kaplan–Meier survival analysis compared to those with duration <5 years. This was not the case when patients were categorized into groups using a threshold of baseline duration of more or less than 1, 2 or 3 years (Fig. 4).

Whether the procedure was performed in the same sitting or by two-stage approach did not impact on 12-month (HR 1.72, 95% CI = 0.47–6.34, *P* = 0.42) or overall AF recurrence (HR 1.40, 95% CI = 0.58–3.35, *P* = 0.46); however, there appeared to be an increase in overall AA recurrence with a two-stage approach (HR 2.24, 95% CI 1.05–4.80, *P* = 0.04). Other factors were not shown to impact on overall AA recurrence.

### Complications

Three patients experienced significant complications (4.5%). These were stroke (*n* = 2) and pseudoaneurysm (*n* = 1), which was treated with thrombin injection. Of these, 2 (1 stroke; 1 pseudoaneurysm) were related to the endocardial stage of the procedure. One patient died of unknown causes 3 months post-endocardial procedure. Given the duration post-procedure, this was considered unlikely related to the procedure. Of the patients who suffered a stroke, 1 patient encountered a consequent delay to the second stage of the procedure; however, the treatment pathways were otherwise not affected.

Other complications were: acute kidney injury (*n* = 1) (mild, not requiring renal replacement therapy) and wound infection (*n* = 1) (methicillin-susceptible staphylococcus aureus organism requiring intravenous antibiotics and lengthened index admission). Five patients (7.5%) experienced clinically significant pericarditis; 3 were mild and 2 required admission for monitoring/treatment. None of these experienced tamponade or required pericardiocentesis. All complications but one resolved; 1 patient who had stroke experienced ongoing residual neurological deficit.

## DISCUSSION

Convergent ablation was effective at treating PsAF, with 81.3% freedom from AF recurrence at 1 year. A high proportion of patients were free of AF and off AADs (68.8%) and the majority were asymptomatic at 1 year (75.0%). Overall freedom from AA recurrence was 69.2% at 12 months. These encouraging results alongside previous findings suggest that convergent ablation is a realistic and effective treatment option for PsAF. AF recurrence increased over time, with 61.5% overall freedom from AF recurrence over follow-up.

Our unselected population was reflective of that encountered in real-world practice and included groups not eligible for the CONVERGE trial [12], including those with previous unsuccessful AF ablation (38.8%) and LVEF ≤40% (19.4%). There was also a high percentage of those with longstanding PsAF >1 year (80.6%), body mass index >30 kg/m^2^ (49.3%) and LA diameter >50 mm (29.9%). Our success rates occurred in a population including many who would be considered to be ineligible or unsuitable candidates for conventional catheter ablation. This treatment may therefore be suitable for many patients who may otherwise have limited options.

Prolonged baseline AF duration was associated with increased AF recurrence at 12 months (HR 1.19, 95% CI = 1.01–1.40, *P* = 0.04) and patients with AF duration >5 years had a shorter time to AF recurrence on Kaplan–Meier analysis (*P* = 0.01). However, AF duration did not impact on overall AF or AA recurrence over the course of follow-up. This suggests that those with prolonged AF duration may experience recurrence earlier than those with shorter duration.

This is informative and should be considered alongside other factors on an individual basis as part of patient selection. Other baseline risk factors were not associated with increased 12-month or overall AF recurrence. Our findings do not suggest that patients with such risk factors should be excluded from consideration for this treatment. Whilst this should be considered alongside other data, convergent ablation may therefore be suitable for a wide range of patients reflected in a real-world setting.

Our study included a combination of same-sitting and staged approaches. Each approach has its merits. In a same-sitting approach, the procedure is completed in a single day and admission, under a single general anaesthetic, which may be more convenient for patients. However, it requires co-ordination for both stages to be performed back to back and involves a longer time under anaesthetic in one sitting. In addition, tissue oedema immediately post-epicardial ablation may make endocardial mapping more challenging. A staged approach may be more feasible to co-ordinate in some centres and allows the maturation of surgical lesions prior to endocardial electroanatomical mapping. In our study, the decision to perform the procedure in 1 or 2 sittings was based on logistics and capacity, rather than patient-specific characteristics.

The type of approach did not impact significantly on AF recurrence in our analysis; however, a same-sitting approach was associated with reduced overall AA recurrence (HR for two-staged approach 2.24, 95% CI 1.05–4.80, *P* = 0.04). The potential mechanism for this is unclear. It is possible that completing the ablation set in close time proximity confers an advantage. However, this is speculative and there has yet to be a direct-controlled comparison between the 2 approaches in the literature. Given the observational nature of our study, these findings highlight an area for potential further study but should not be used to favour one approach or other.

During endocardial ablation, 14% received LA roof line and 6% received posterior box ablation lesions. Whilst it is counter-intuitive that patients would require these after receiving extensive epicardial posterior wall ablation, this is likely to reflect anatomical limitations. In our experience, the attachments of the pericardial reflections can limit the extent of possible epicardial ablation in some cases, particularly in the upper portion of the posterior LA. Therefore, in a proportion of patients, a ‘touch-up’ is required during the endocardial stage to complement and adjunct the epicardial ablation. This is often less extensive than would be required if posterior wall silencing was solely performed by endocardial ablation. In our cohort, in patients with PsAF duration under 1 year, none were felt to require additional ablation lesions above PVI and CTI line during the second stage of the procedure.

The overall rate of significant complications was 4.5%. This needs to be carefully weighed against potential benefit in the individual when selecting patients. In previous studies, which reflect early experiences, complication rates have ranged from 5.7% to 12.9% [9, 11, 13, 14]. Procedural safety is an important consideration in the adoption of a procedure. There is a learning experience with a new procedure and safety is expected to further improve as experience grows and as technique is refined. For example, advancements such as using sub-xiphoid approach to minimize the risk of hernia, the addition of oesophageal temperature monitoring, saline irrigation and refinement of the epicardial lesion set have already been employed to improve safety based on learning from early experiences [15]. In a single-centre analysis, transition from transdiaphragmatic to sub-xiphoid approach resulted in a significant reduction in complications from 23% to 3.8% [10].

The rate of pericarditis in our study was 7.5%. Two patients required admission for treatment but no patients required procedural intervention. Following early experiences of convergent ablation, practices have evolved to include prophylactic use of steroids and/or colchicine to combat pericardial effusion [15].

There was a high incidence of AT and AFL following the blanking period (30.8%) in our study, including those who experienced both AT and AF recurrence. This may reflect the degree of scar created during the convergent procedure. It is anticipated that these would be effectively treated with DCCV or repeat ablation that may be less challenging than PsAF catheter ablation. In our study, 13 of the 20 patients experiencing AT/AFL were subsequently successfully treated with ablation or DCCV.

Whilst direct comparisons are difficult due to the observational nature of our study, our overall success rates are consistent with outcomes from previous reports of the convergent procedure and compare favourably to many reported success rates for catheter ablation [4, 5]. Gulkarov *et al.* [13] reported a series of 31 patients undergoing convergent ablation, with 29% experiencing AF recurrence at 1 year and 48% at 2 years. Another recent study found that 60.5% of 43 patients were AF free at 1 year post-convergent ablation [11]. In the CONVERGE-IDE, 67.7% of patients were free from all AA at 12 months post-convergent ablation, with superior effectiveness to catheter ablation (67.7% vs 50.0%, *P* = 0.036) [12].

Another study involving 113 patients, of whom 82% had implantable loop recorders for more complete continuous rhythm monitoring, found 12-month freedom from arrhythmia to be 53% [10]. In this study, the mean burden of AA in those with recurrence was <5%, suggesting that even in those who had recurrence, there may be significant burden reduction and consequent improvement in symptoms and QOL. Patients undergoing AF rhythm-control treatment may experience QOL benefits despite AF recurrence as long as arrhythmia burden remains low [16]. Furthermore, 74.0% of patients undergoing convergent ablation in the CONVERGE trial had a 90% or greater reduction in AF burden at 18 months [12].

There are numerous mechanisms through which convergent ablation may be successful. Creating transmural lesions may be crucial to avoid the recurrence of AF due to epicardial breakthrough waves despite endocardial layer isolation. In addition to electrical isolation, epicardial ablation may result in autonomic modulation through ablation of epicardial neural network and fat tissue. In addition, debulking of the LA mass play a role in reducing AF through substrate reduction [17].

The multi-disciplinary Heart Team was crucial to the setup of our convergent ablation program and to success in our study. This facilitates appropriate patient selection, preprocedural optimization, appropriate procedure stage co-ordination and homogenization of follow-up strategies. This delivers a harmonized approach. Communication between the cardiac centre and local hospital is important for troubleshooting and appropriate long-term follow-up. This approach is vital for departmental learning and development when undertaking a relatively novel technique.

PsAF places a significant strain on healthcare resources. Treating PsAF effectively may reduce hospitalizations, repeat procedures and be cost-effective. We did not review cost-effectiveness in our study. However, a US micro-simulation analysis found convergent ablation to have superior cost-effectiveness to both catheter ablation and medical management over 5 years, especially for medium- and high-risk patients, based on observational data. Those undergoing convergent ablation underwent an average of 1.1 procedures compared to 1.65 in those undergoing catheter ablation. The higher initial procedure cost with convergent ablation was offset by a reduction in repeat ablations and AF-associated events, with an average saving of over $8000 in high-risk patients [18].

### Further studies

There is a growing body of evidence supporting the efficacy of convergent ablation for PsAF. Further research will evaluate longer-term efficacy and further work on the mechanisms underlying procedural success and the adjunctive role of left atrial appendage exclusion would provide useful advancements to the knowledge base.

### Limitations

Our study is a retrospective, observational study with no comparator group and should be interpreted in this context. Conclusions should be further evaluated alongside larger-scale, randomized, prospective data. Catheter ablation strategy was varied within this study; this however reflects real-world practice.

As this was an observational study, the follow-up and approach to continuous rhythm monitoring were not uniform throughout follow-up for all patients. Brief paroxysmal and asymptomatic episodes may therefore have been under-detected. When patients reported symptoms and ECG was normal, patients were referred for continuous rhythm monitor if not already performed. It is felt that this approach would have detected the majority of clinically significant arrhythmias and is similar to methods used in other studies; however, there is a risk of under-detection of AF episodes inherent in this approach.

## CONCLUSIONS

Convergent ablation was effective at treating PsAF in our study, with encouraging rates of freedom from AF in a challenging population. Convergent ablation is a promising potential option for many patients and applicable to a real-world population.

## Funding

This research did not receive any specific grant from funding agencies in the public, commercial or not-for-profit sectors.

## ETHICS APPROVAL

Institutional approval was granted from our institution’s clinical effectiveness unit.

## PERMISSION TO REPRODUCE MATERIAL FROM OTHER SOURCES

Permission is obtained from Atricure to use relevant images (Fig. 1—see figure legend).

**Conflict of interest:** Outside of the submitted work: Nilanka N. Mannakkara, Bradley Porter, Nicholas Child, Vishal S. Mehta, Mark K. Elliott and Justin Gould have received educational fellowship funding from Abbott; Baldeep S. Sidhu has received funding by a project grant from NIHR and has received speaker fees from EBR systems; Jaswinder S. Gill has also received project funding from Rosetrees Trust; Christopher A. Rinaldi has received research funding and/or consultation fees from Abbott, Medtronic, Boston Scientific, Spectranetics and Microport; Christopher Blauth has consulted for New Cardioplegia Solutions and as a proctor for Atricure; and Jaswinder S. Gill has received research funding from Abbott and lecture honoraria from Atricure. The other authors report no conflict of interest.

### Data Availability Statement

Data underlying this article cannot be shared publicly and are not available.

### Author contributions

**Nilanka N. Mannakkara:** Data curation; Formal analysis; Investigation; Methodology; Project administration; Writing—original draft; Writing—review & editing. **Bradley Porter:** Data curation; Investigation; Methodology; Writing—review & editing. **Nicholas Child:** Data curation; Investigation; Methodology; Writing—review & editing. **Baldeep S. Sidhu:** Writing—review & editing. **Vishal S. Mehta:** Writing—review & editing. **Mark K. Elliott:** Writing—review & editing. **Justin Gould:** Writing—review & editing. **Shahada Ahmed:** Writing—review & editing. **Reza Razavi:** Writing—review & editing. **Christopher A. Rinaldi:** Writing—review & editing. **Christopher Blauth:** Writing—review & editing. **Jaswinder S. Gill:** Conceptualization; Investigation; Methodology; Project administration; Supervision; Writing—review & editing.

### Reviewer information

European Journal of Cardio-Thoracic Surgery thanks Francesco Onorati and the other, anonymous reviewer(s) for their contribution to the peer review process of this article.

## ABBREVIATIONS

AA: Atrial arrhythmia
AADs: Anti-arrhythmic drugs
AF: Atrial fibrillation
AFL: Atrial flutter
AT: Atrial tachycardia
CI: Confidence interval
CTI: Cavotricuspid isthmus
DCCV: Direct current cardioversion
HR: Hazard ratio
ECG: electrocardiogram
LA: Left atrium
LVEF: Left ventricular ejection fraction
PsAF: Persistent atrial fibrillation
PVI: Pulmonary vein isolation
SD: Standard deviation
